# Influence of Oral Appliances for Mandibular Advancement on Dentitions Using a Strain Gauge Analysis: A Pilot Study

**DOI:** 10.1155/2017/9097305

**Published:** 2017-04-03

**Authors:** Hiroshi Ueda, Yu Matsumura, Atsushi Horihata, Cynthia Concepcion, Koji Iwai, Kotaro Tanimoto

**Affiliations:** Department of Orthodontics and Craniofacial Developmental Biology, Hiroshima University Graduate School of Biomedical Sciences, Hiroshima, Japan

## Abstract

*Introduction*. This study aimed to evaluate the influence of oral appliances (OAs) on dentition using a strain gauge analysis.* Materials/Methods*. Eight volunteers, who were mild snorers, participated in this study. OAs were individually constructed, and advancement was defined as two-thirds of the maximum mandibular advancement. Strain gauges were mounted on the right first molar and central incisor of both the upper and lower arches. After OA use, two measurement sessions (short- and long-term) were performed.* Results*. Compressive strain on the labial surface was significantly larger than the stretching strain on the lingual surface on U1. On L1, the stretching strain on the labial surface was significantly larger than the compressive strain on the lingual surface. Comparing the upper and lower teeth, the stretching strain was significantly greater on L1 than on U1 in both test sessions. Moreover, the stretching strain was significantly larger on U6 than on L6.* Conclusion*. OA side effects, such as forcing on the incisors, might be repeated every night. In this way, permanent occlusal changes, such as labial tipping of L1, may occur, followed by lingual tipping of U1 and buccal and lingual movements of the U6 and L6, respectively.

## 1. Introduction

Oral appliances (OAs), which aim to enlarge the upper airway by repositioning the mandible forward [1–3], have been established as a lifelong treatment tool for primary snoring and mild-to-moderate obstructive sleep apnea (OSA). OSA is a respiration-related complication characterized by repetitive partial or complete obstruction of the upper airway during sleep. OSA is associated with increased morbidity and mortality from cardiovascular events, and descriptive lists of OSA complications in adults have been previously reported [4, 5].

OAs are also used by patients with moderate-to-severe OSA when they refuse nasal continuous positive airway pressure (nCPAP) therapy [6, 7]. Compared to nCPAP devices, OAs are relatively small and easy to wear; however, recent studies in OSA patients report that long-term use of OAs was accompanied by considerable occlusal changes, such as decreases in both the overbite and overjet and forward movement of the mandibular molars [8–18]. Moreover, Rose et al. reported that a temporary bite change occurs in most patients the morning after removal of the appliance, and permanent occlusal changes have been observed after long-term treatment with OAs in individual cases [19]. Martínez-Gomis et al. estimated the occlusal contact in the intercuspal position by using occlusal registration strips and showed that the number of occlusal contacts was significantly decreased in patients undergoing OA therapy [20]. The side effects of OAs on dentition have not been investigated in detail; therefore, it is important to analyze dental and occlusal changes associated with the use of OAs.

The aim of the present study was to evaluate the influence of wearing OAs on dentition using a strain gauge analysis.

## 2. Materials and Methods

### 2.1. Subjects and Oral Appliance

The Ethics Committee of Hiroshima University Hospital approved the study protocol, and informed consent was obtained from each subject prior to the experiment. The study subjects consisted of 8 adults (4 men and 4 women; mean age, 28.3 ± 1.7 SD years) selected from volunteers in the Department of Orthodontics at Hiroshima University Hospital who snore mildly. The inclusion criteria for the subjects were as follows: (1) normal horizontal and vertical skeletal relationships, (2) no severe malocclusions, and (3) no complaints of a temporomandibular joint and muscle (TMJ) disorder.

**Figure 1:**
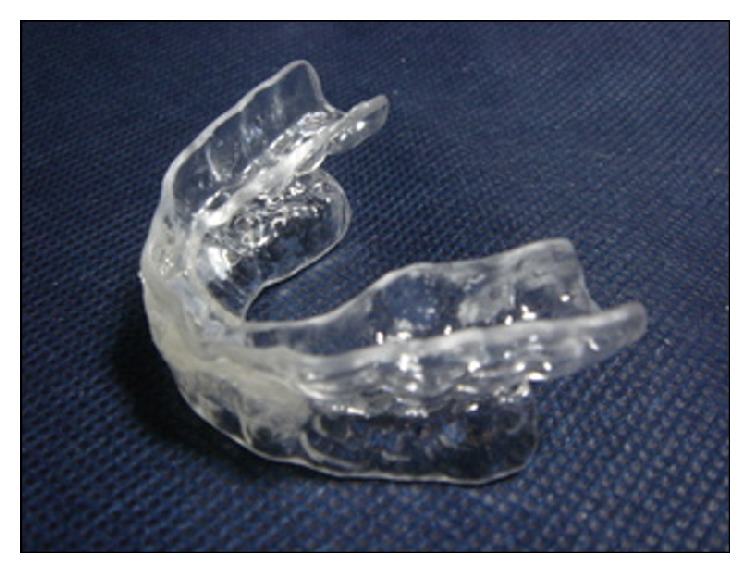
An oral appliance.

One-piece custom OAs were made for all subjects after obtaining impressions of the maxillary and mandibular dentitions. OAs were constructed from a 0.75 mm thick acrylic resin that provided full occlusive coverage of the teeth. The initial advancement was defined as two-thirds of the maximum mandibular advancement (6.2 ± 0.9 mm) with a 2-3 mm vertical opening at the anterior teeth (Figure 1).

### 2.2. Recording System

**Figure 2:**
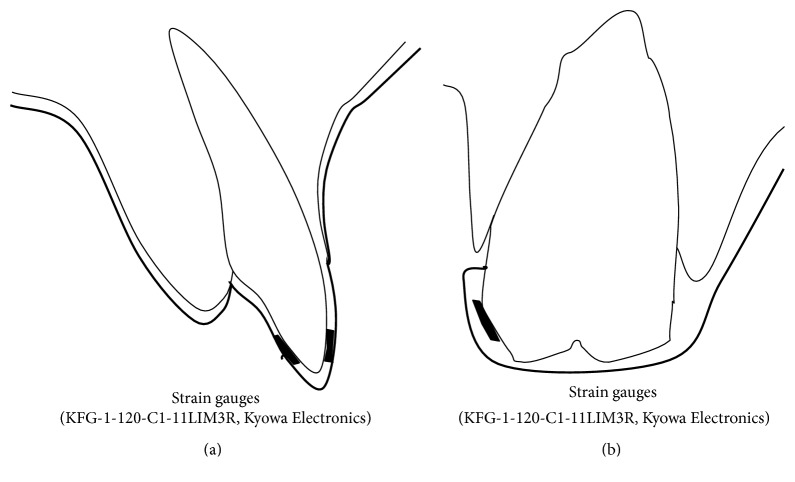
Placement of strain gauges on (a) the central incisor and (b) the first molar. The thick line indicates the OA surface.

**Figure 3:**
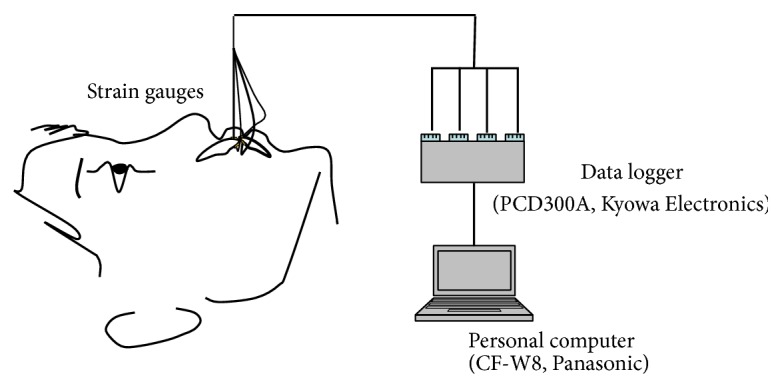
Schematic block diagram of the measuring strain system.

Strain gauges (KFG-1-120-C1-11LIM3R, Kyowa Electronic Instruments Co., Tokyo, Japan; 2.4 mm × 4.8 mm × 13 *μ*m) were mounted at the right first molar and central incisor regions of the upper and lower oral appliances. To measure strain on the central incisors, we chose one labial-facing incisor and one lingual-facing incisor. To measure the first molar, the strain gauge was placed on the buccal side (Figures 2(a) and 2(b)). The strain gauges were bonded to the inside of the oral appliance with cyanoacrylate adhesive (CC-33A, Kyowa Electronic Instruments Co., Tokyo, Japan) to avoid the influence of humidity. Strain was detected using a dynamic data logger (PCD300A, Kyowa Electronic Instruments Co., Tokyo, Japan) to which the wires were connected. The data were transferred to a computer (CF-W8, Panasonic, Osaka, Japan) that used data analysis software (DAS-100A, Kyowa Electronic Instruments Co., Tokyo, Japan) (Figure 3).

### 2.3. Recording Procedure

In this experiment, two sessions were conducted, one for a short period and another for a longer period with the use of the OA on the same day. The short-term and long-term recordings were carried out for 60 seconds and 60 minutes, respectively. During measurement, the participants were requested to relax their jaw muscles and maintain a supine position on the bed, assuming a sleep posture. All measurements were performed twice.

Data are presented as the mean ± standard deviation and were analyzed using an analysis of variance (ANOVA) and pairwise comparisons (Scheffe's test). *P* values less than 0.05 were considered significant. Statistical analysis was carried out using Statcel 2 (OMS, Inc., Tokorozawa, Japan).

## 3. Results

### 3.1. Short-Term Recordings

The stretching strain on the upper central incisor was significantly less than on the lower central incisor (Table 1). The compressive strain on the upper central incisor was significantly larger (*P* < 0.01) than the stretching strain on the same tooth.

The compressive strain was slightly larger on the lower central incisor than on the upper incisor. In comparing both stretching and compressive strain, the difference was not found to be statistically significant on the lower central incisor (Table 1).

With respect to the first molar, there were no notable differences in stretching strain between the upper and lower teeth. The compressive strain trend was marginally greater on the upper first molar, even though none of these results were statistically significant (Table 2).

### 3.2. Long-Term Recordings

Stretching strain was significantly less on the upper central incisor than on the lower central incisor (*P* < 0.01). In contrast, compressive strain was moderately larger on the upper incisor than on the lower incisor (Table 1).

Assessing the contrasts between stretching and compressive strains, the upper central incisor stretching strain on the lingual side was significantly less than the compressive strain on the labial side (*P* < 0.05). However, for the lower central incisor, stretching strain on the labial side was significantly greater than the compressive strain on the lingual side (*P* < 0.05) (Table 1).

On the posterior sector, the mean stretching strain on the upper molar tended to be greater compared to the compressive strain on the upper molar, based upon a long-term recording (Table 2). In contrast, the mean compressive strain of the lower molar was considerably larger than the mean stretching strain, although no significant difference was found (Table 2). In addition, the stretching strain of the upper first molar was significantly larger (*P* < 0.05) than that of the lower molar, based upon long-term recordings (Table 2).

## 4. Discussion

In the present study, we investigated the influence of one-piece OAs on the upper and lower incisors and first molars using short-term and long-term recordings. On the upper central incisor, the compressive strain on the labial side was significantly larger than the stretching strain on the lingual side. In contrast, the stretching strain on the labial side was significantly larger than the compressive strain on the lingual side for the lower central incisor.

Generally, the design of OAs for patients with OSA is based on functional orthodontic appliances that enhance mandibular growth in developing patients with a small and/or distally located mandible. The construction bite indicated that the initial mandibular advancement was two-thirds of the maximum mandibular forward position with a 2-3 mm vertical opening at the anterior teeth. The force induced by the mandible, which opposes the construction bite, has considerable influence on the labial direction of the lower incisors and the lingual direction of the upper incisors. These forces are proportional to the amount of the advancement of the mandible. Cohen-Levy et al. used a pressure transducer system with OA in OSA patients and measured forces created by progressive mandibular advancement for each step of 1 mm. They found that the mean force was 1.18 N/mm, which exhibited an almost linear evolution [21].

Moreover, stretching strain on the lower central incisor was significantly greater than that on the upper incisor in short-term and long-term recordings. One explanation for the difference in strain between the upper and lower incisors is that the size of the incisor crowns and the surface dimension of a whole tooth body are different. The net amount of force that is required for tooth movement mainly depends on the root surface [22]. Interestingly, in our previous study, the mobility of the lower incisors immediately after nocturnal use of OA was the highest among all teeth, followed by the upper incisors [23].

**Figure 4:**
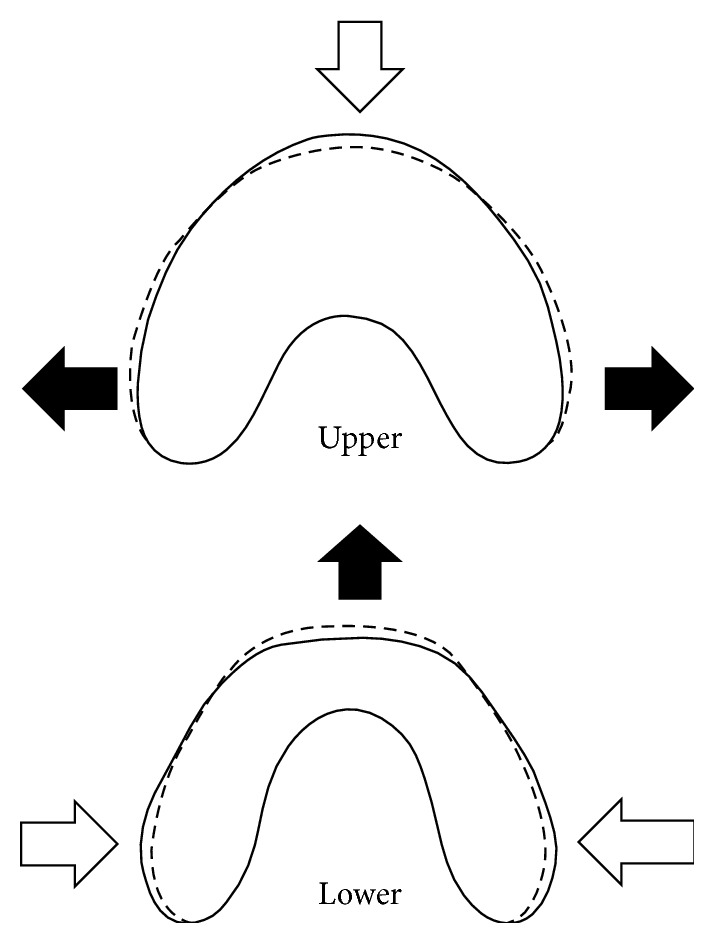
Illustration of the force direction on the OAs. Black arrow, outside direction; white arrow, inside direction.

Notably, the present study found a significantly greater stretching strain on the upper first molar than on the lower one in the buccal direction compared to the compressive strain. A previous study has described similar results for the upper dental arch. De Almeida et al. reported an increase in maxillary canine and molar widths after a 5-year use of OA, although the sample was in a small section of patients with Class II Division 2 malocclusions [24]. We speculate that the maxillary incisors are retroclined with the use of OAs, possibly causing an enlargement of the maxillary arch length, especially the lateral teeth region, which results in an increase in the intermolar distance (Figure 4).

This study has certain limitations, including its small sample size and the limited positions of strain gauges on the OAs. However, we hope our results support further investigations that focus on the effects of various directions of adverse forces that are induced by OAs on dentitions in a larger number of cases of patients who snore or experience mild OSA.

## 5. Conclusions

For the upper central incisor, the compressive strain on the labial side was significantly larger than the stretching strain on the lingual side. In contrast, for the lower central incisor, the stretching strain on the labial side was significantly larger than the compressive strain on the lingual side. Stretching strain on the lower central incisor was also significantly greater than that on the upper incisor. Moreover, stretching strain on the upper first molar was found to be significantly greater than that on the lower one in the buccal direction. Therefore, OA side effects, such as forcing on the incisors, might be repeated every night. In this way, permanent occlusal changes such as labial tipping of the incisors in the lower dentition may occur, followed by lingual tipping of the upper incisors and buccal and lingual movements of the upper and lower first molars, respectively.

## Figures and Tables

**Table 1:** Comparison of the strain on the incisors.

Recording period	Mean ± SD			
	Site	Stretching strain	Compressive strain	Stretching versus compressive strains
Short	Upper incisor	2.5 ± 5.8^*∗∗*^	17.7 ± 7.9	*∗∗*
	Lower incisor	25.3 ± 14.6^*∗∗*^	26.5 ± 33.7	NS
Long	Upper incisor	25.7 ± 33.9^*∗∗*^	67.8 ± 44.6	*∗*
	Lower incisor	119.7 ± 2.6^*∗∗*^	60.6 ± 52.4	*∗*

Stretching strain: upper-lingual side; lower-labial side.

Compressive strain: upper-labial side; lower-lingual side.

^*∗∗*^ *P* < 0.01.

^*∗*^ *P* < 0.05.

NS = not significant.

**Table 2:** Comparison of the strain on the first molars.

Recording period	Mean ± SD			
	Site	Stretching strain	Compressive strain	Stretching versus compressive strains
Short	Upper molar	33.2 ± 26.3	31.1 ± 18.2	NS
	Lower molar	37.8 ± 33.3	21.7 ± 21.4	NS
Long	Upper molar	62.6 ± 27.0^*∗*^	46.3 ± 35.3	NS
	Lower molar	37.5 ± 19.4^*∗*^	60.5 ± 49.0	NS

^*∗*^ *P* < 0.05.

NS = not significant.
